# Unusual Case of Inflammatory Myofibroblastic Tumor in Maxilla

**DOI:** 10.1155/2013/876503

**Published:** 2013-04-16

**Authors:** Jaana Rautava, Tero Soukka, Esko Peltonen, Petri Nurmenniemi, Markku Kallajoki, Stina Syrjänen

**Affiliations:** ^1^Department of Oral Pathology and Oral Radiology, Institute of Dentistry, University of Turku, Lemminkäisenkatu 2, 20520 Turku, Finland; ^2^Cell Biology Program, Research Institute, Hospital for Sick Children, University of Toronto, Room 7142, 555 University Avenue, Toronto, ON, Canada M5G 1X8; ^3^Department of Oral and Maxillofacial Surgery, Institute of Dentistry, University of Turku, Lemminkäisenkatu 2, 20520 Turku, Finland; ^4^Department of Pathology, University of Turku, Kiinanmyllynkatu 4-8, 20520 Turku, Finland

## Abstract

Inflammatory myofibroblastic tumor (IMT) is a rare lesion found mostly in children and young adults and originates from the lung, abdominopelvic region, and retroperitoneum. Clinical manifestations of IMT or imaging are nonspecific and diagnosis is based on histopathological and immunohistochemical findings. Minority of all IMTs will metastasize. IMT in the oral cavity is an extreme rarity and this is a first case report of IMT in maxilla causing delayed tooth eruption and multiple cervical root resorption with an 11-year-old child. The IMT reported here was positive for smooth muscle actin, vimentin, and anaplastic lymphoma kinase (ALK1) with immunohistochemistry. Only three IMTs of the jaws have been reported so far and none of them had delayed root eruption and tooth resorption. This unusual case of IMT in a child was also ALK1- positive supporting neoplastic origin of her tumor. The case presented here underscores the importance of histopathological examination of the tissue found in any root resorption especially in the case of multiple resorptions.

## 1. Introduction

Inflammatory myofibroblastic tumor (IMT, also known as inflammatory pseudotumor, plasma cell granuloma, inflammatory fibrosarcoma, fibrohistiocytoma, etc.) is composed of myofibroblastic cells with inflammatory infiltrate [1, 2]. It is a rare lesion which has a predilection for children and young adults. Most reported cases have been from the lung, abdominopelvic region, and retroperitoneum. Trauma and viral infections such as herpes simplex virus type 8 (HSV-8) and Epstein-Barr virus (EBV), as well as autoimmune reactions, have been suggested as etiological factors. Still, exact etiology remains elusive but more recently neoplastic origin has been suggested as the most likely candidate [3]. IMT may mimic a malignant process. A recent review of the literature reported 22 cases of oral IMT and most of them were observed on buccal mucosa and only three occurred in the jawbones [4]. Since then two more cases of oral IMTs have been reported, one in the tongue and one as a soft tissue mass of the mandibular region [5, 6]. 

## 2. Case Report

**Figure 1:**
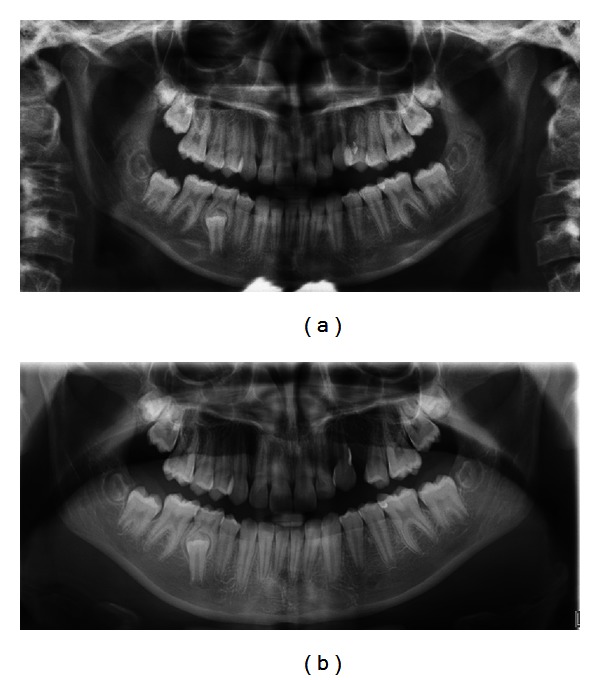
(a) The first panoramic image shows an osteolytic area on the marginal bone border between 22 and 24. There is a deep vertical bone lesion on the mesial side of 24. It is challenging to see resorption on the coronal part of the roots. (b) Panoramic image three months after the extraction of 24. The healing of the extraction region is in progress. In the region 22-23, the osteolytic area has remained the same compared to the earlier image.

An 11-year-old previously healthy girl with no medications or allergies had complicated eruption of the left maxillary canine tooth 23 and first premolar 24. A panoramic tomography showed a large resorption of the mesial side of the root of 24 (Figure 1(a)). There was no previous history of trauma to the area. Clinically, marginal gingivitis around the teeth 22–24 was found, both labially and buccally, in addition to external resorption of 24. Six-to-eight millimeter deep gingival pockets were detected adjacent to the teeth 23 and 24. Patient complained of soreness of the area. According to an orthodontic specialist, there was a lack of space and tooth 24 was extracted and granulation tissue was enucleated. At this point, no tissue was obtained for histopathological diagnosis. Twelve weeks later, a significant bone defect was observed and the position of 23 was still in infraocclusion and the patient was referred to the Department of Oral and Maxillofacial Surgery, Turku University Hospital (TUH), Turku, Finland. 

At TUH, the bone defect was confirmed. No swelling, deep gingival pockets, or mobility of the teeth in this region were detected on the clinical examination. The adjacent teeth were vital, but there was a slight decrease in vitality related to the second premolar and first molar. The gingiva in the region showed bluish colouring. 

**Figure 2:**
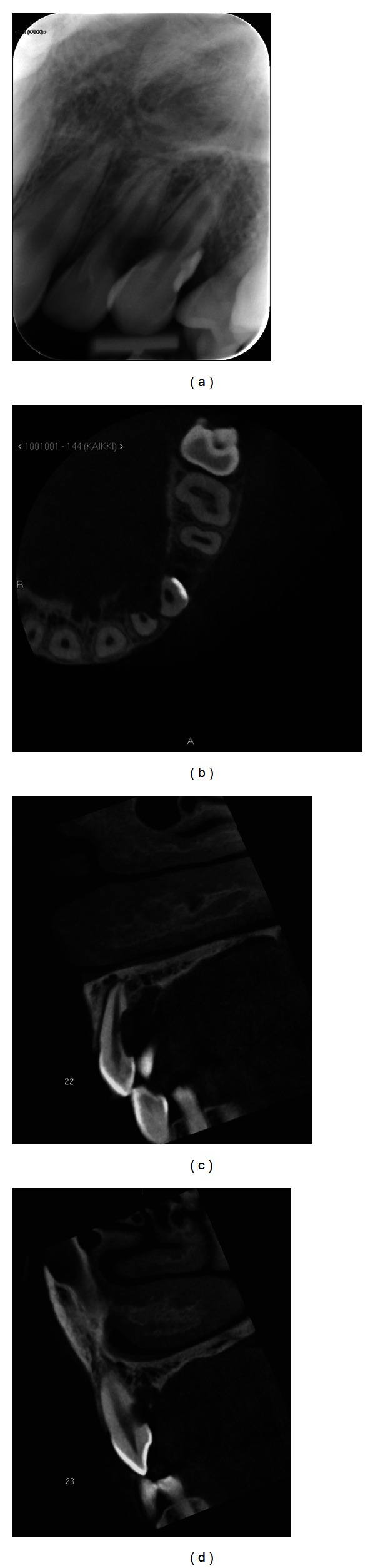
(a) The root resorption is visible in the intraoral X-ray of 22-23. (b) CBCT axial slice above the marginal bone line. A 6 × 10 mm bone destruction, which is sharply lined, is visible in the region 22-23 on the palatinal side. The roots of the teeth 22-23 have resorption on the palatinal sides. (c) CBCT sagittal slice presenting the root of 22. On palatinal side of the root, there is a 2 × 4 mm deep resorption defect. Lesion on palatinal bone structure can also be seen. (d) CBCT sagittal slice of 23.

Panoramic and intraoral X-rays revealed external resorption in the roots of left maxillary lateral incisor and canine teeth. An osteolytic area in the same region (teeth 22-23) was detected consistently with an image obtained three months earlier (Figure 1(b)). Furthermore, cone beam tomography revealed a cystic, osteolytic area in the left palatal side of the roots from lateral incisor to first premolar in the left side (Figure 2). The palatal cortex was perforated. 

The clinical and radiographic pictures were nonspecific. Resorption of multiple teeth and the perforation of palatal cortex suggested locally aggressive lesions. The differential diagnoses included aggressively behaving odontogenic tumors, nodular fasciitis, and malignant lesion. Ameloblastoma is the most common aggressive benign odontogenic tumor but it is rare in children. The resorption of adjacent teeth is common with ameloblastoma but it often presents as a swelling of jaw. Furthermore, the image in panoramic tomography was not lobulated as often found with ameloblastomas. Nodular fasciitis is a benign, reactive process with rapid growth that mimics malignant soft tissue sarcomas. However, also nodular fasciitis is usually diagnosed in adults. Nodular fasciitis is often superficially located at soft tissue mass and is associated with pain. 

The likely possible malignancies under consideration were malignant soft tissue sarcomas, malignant bone tumors, and Burkitt's lymphoma. Of the malignant soft tissue sarcomas, rhabdomyosarcoma (especially the embryonic variant) is a relatively common malignant soft tissue tumor in children. Fibrosarcoma is a malignant mesenchymal tumor of fibroblasts that may arise in the jaws, especially the mandible. It usually affects young adults but has been reported also in children. Malignant bone tumors constitute the 6th most common group of malignant neoplasms in children, with osteosarcoma being the most common pediatric bone tumor and Ewing's sarcoma family of tumors being the second. Both may result in an osteolytic image of the jaws in radiographs. Burkitt's lymphoma is related to EBV. It typically affects children and maxilla and may cause tooth displacement or loss, gingival expansion, and pain. 

The lesion was treated by enucleation and curettage and the beyond-repair damaged teeth with resorption were extracted and sent for histopathologic examination to the Department of Oral Pathology and Oral Radiology, Institute of Dentistry, University of Turku, Turku, Finland.

**Figure 3:**
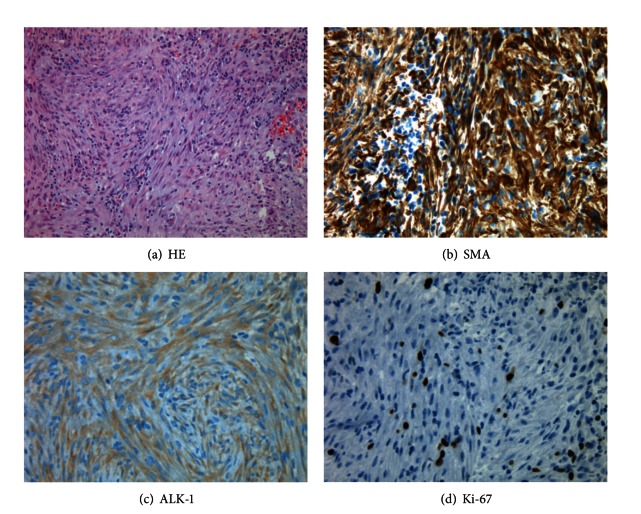
(a) Hematoxylin-eosin staining showed dense aggregates of spindle cells in storiform architecture with scattered lymphocytes. (b) Immunohistochemical staining is strongly positive with smooth muscle actin and (c) with ALK (d) while Ki-67 positivity was low.

Histopathological investigation revealed a compact cellular spindle cell proliferation with collagenous stroma with storiform architecture (Figure 3(a)). The proliferation started directly under the oral mucosal epithelium continuing throughout the tissue samples. The uniform spindle cells contained ovoid nuclei with rather pale eosinophilic cytoplasm. Only mild nuclear pleomorphism was seen. Mitotic activity was low as detected with Ki-67 level (Figure 3(d)). Inflammatory component was minor with single or small groups of lymphocytes scattered around the spindle cell proliferation. With immunohistochemistry (IHC), the tumor tissue was positive for smooth muscle actin (SMA), vimentin, and anaplastic lymphoma kinase (ALK) (Figures 3(b)-3(c)). Calponin, myoglobin, and HHF35 antibodies showed moderate staining in myofibroblasts. Immunostaining of CD34 showed intense vascularisation of the whole tumor. There was no immunoreactivity for S-100, pancytokeratin, desmin, or Myf4. With PCR using the DNA extracted from the paraffin blocks, no HSV-8 or EBV was detected. The final diagnosis was inflammatory myofibroblastic tumor (IMT).

At the department of pediatric oncology, the patient was clinically examined and no symptoms or clinical signs of disease were detected locally or distantly. Furthermore, the complete blood count with C-reactive protein (CRP) and erythrocyte sedimentation rate were within normal limits. Whole body magnetic resonance imaging (MRI) or bone scintigraphy (^99m^TcHDP) did not reveal signs of occult disease. After three years of followup, there was no recurrence.

## 3. Discussion

To our knowledge, only three intrabony IMTs of the oral cavity have been reported but none of them had delayed tooth eruption and root resoption [3]. The ALK (chromosome band 2p23) gene has been implicated in the pathogenesis of IMT supporting neoplastic origin of tumor [3]. Approximately 50% of all IMTs have been associated with ALK positivity. Most of the previous oral IMT cases have been reported to be ALK negative [4]. Indeed, a question has been raised whether ALK-positive IMTs are a distinct clinicopathologic entity with neoplastic origin with more aggressive behavior and even potential for malignancy. The present case was ALK positive, but within the follow-up time of three years, the tumor has not shown any signs of recurrence.

IMT can present with variable clinical manifestations. Therefore, clinical manifestations of IMT or radiographic images have poor specificity to make a diagnosis, as was shown also with the present case. Diagnosis of IMT is based on histopathological and immunohistochemical findings according to The World Health Organisation (WHO) classification of soft tissue tumors [2]. Histomorphology of IMT has a high degree of overlap with other spindle cell lesions. Differential diagnosis of malignancies such as fibrosarcoma or spindle cell carcinoma is difficult without any immunohistochemistry. Also gingival or periodontal inflammation might easily mask the typical features of the lesion. Collectively, most extrapulmonary IMTs display immunohistochemical reactivity for vimentin, SMA, muscle specific actin, and desmin, but not for S-100 [2]. 

IMT is classified as a benign lesion but local aggressiveness may be associated with chromosomal aberrations [3]. Aggressive behavior has been associated with high degree of cellular atypia with increased mitotic figures, multinodularity, and presence of ganglion-like cells [7]. In addition, IMTs with ALK positivity, elevated Ki-67 proliferative index (up to 10%), DNA aneuploidy, or oncogenic protein overexpression (p53, bcl-2) exhibit increased risk for recurrence [7]. Among nonoral IMTs, recurrence is seen in 19%–25% of all patients with average of 6 months' time period and in 15% of pediatric patients only. Metastasis is rare and is detected in less than 5% of cases. Moreover, there is a small possibility of malignant transformation to sarcoma. According to the recent literature, oral IMTs seem to run a more favorable clinical course with no likelihood of recurrence or malignant transformation [4]. 

For oral IMTs which are most likely to display local aggressiveness but not metastasize, management with complete surgical resection and 10-year postoperative reassessment has been suggested [8]. With nonoral extrapulmonary IMTs, where aggressive behavior is more likely to occur, recent reports have found steroid treatments combined with surgery effective at least for pediatric cases. Currently, on-going research attempts to find a molecular therapy against diseases with ALK-driven pathways [9].

The cause of external cervical root resorption is poorly understood. Etiological factors from dental treatment (or trauma) to bruxism and developmental defects have been suggested. It is well established that certain neoplasms or cysts can cause tooth resorption. Multiple idiopathic cervical root resorptions are most frequently seen in young females. In the present case, we suggest that the resorptions were most likely caused by the tumor. Interestingly, a recent publication reported an IMT of a maxillary sinus which caused pulp necrosis of maxillary teeth [10]. 

## 4. Conclusion

Maxillary IMTs are extremely rare. Furthermore, oral IMTs with ALK positivity present the minority of the reported cases. The case presented here underscores the importance of histopathological examination of the tissue found in any root resorption cavities especially in the cases with multiple resorptions.
